# Analysis of Antibiotic-Resistant and Virulence Genes of *Enterococcus* Detected in Calf Colostrum—One Health Perspective

**DOI:** 10.3390/ani13121900

**Published:** 2023-06-07

**Authors:** Sandra Cunha, Carla Miranda, Ângela Martins, Rúben Soares, Manuel Maia, Filipe Silva, Gilberto Igrejas, Patrícia Poeta

**Affiliations:** 1 Microbiology and Antibiotic Resistance Team (MicroART), Department of Veterinary Sciences, University of Trás-os-Montes and Alto Douro, 5000-801 Vila Real, Portugal; al62192@utad.eu (S.C.); al70477@alunos.utad.pt (R.S.); al60199@alunos.utad.pt (M.M.); ppoeta@utad.pt (P.P.); 2 Associated Laboratory for Green Chemistry (LAQV-REQUIMTE), University NOVA of Lisbon, 1099-085 Caparica, Portugal; gigrejas@utad.pt; 3 Toxicology Research Unit (TOXRUN), University Institute of Health Sciences, Advanced Polytechnic and University Cooperative (IUCS-CESPU), 4585-116 Gandra, Portugal; 4 Department of Zootechnics, University of Trás-os-Montes and Alto Douro, 5000-801 Vila Real, Portugal; angela@utad.pt; 5 Veterinary and Animal Research Centre (CECAV), University of Trás-os-Montes and Alto Douro, 5000-801 Vila Real, Portugal; fsilva@utad.pt; 6 Associate Laboratory for Animal and Veterinary Science (AL4AnimalS), University of Trás-os-Montes and Alto Douro, 5000-801 Vila Real, Portugal; 7 Department of Veterinary Sciences, University of Trás-os-Montes and Alto Douro, 5000-801 Vila Real, Portugal; 8 Department of Genetics and Biotechnology, University of Trás-os-Montes and Alto Douro, 5000-801 Vila Real, Portugal; 9 Functional Genomics and Proteomics Unit, University of Trás-os-Montes and Alto Douro, 5000-801 Vila Real, Portugal

**Keywords:** antimicrobial resistance, bovine colostrum, *Enterococcus*, virulence profile

## Abstract

**Simple Summary:**

Enterococci are among the most responsible agents for nosocomial infections and are globally prevalent antibiotic-resistant microorganisms. The risk of calves being fed colostrum contaminated with these bacteria or antimicrobial-resistant bacteria, leading to the colonization of their gastrointestinal tract, is a concern of public health. The objective of this study was to investigate whether bovine colostrum can act as a reservoir and vehicle for the dissemination of antibiotic-resistant *Enterococcus* spp. via the food chain. The sensitivity to 14 antibiotics using the disk diffusion method, as well as the presence of antibiotic-resistant genes and virulence genes, were analyzed in calf colostrum samples. *E. faecalis*, *E. faecium* and *E. gallinarum* were identified in the colostrum samples. The results demonstrated a very high percentage (92.1%) of isolates classified as multidrug-resistant (≥3 antimicrobial classes). Additionally, 52% of the isolates showed the presence of ≥4 virulence genes. *E. faecium* was most likely to carry erythromycin and tetracycline resistance genes, as well as virulence genes. This study revealed that colostrum serves as a reservoir and/or vehicle for the spread of antibiotic resistance and virulence genes. These results have to be analyzed in a One Health perspective to better combat this spread to humans, other animals and the environment.

**Abstract:**

*Enterococci* are considered among the most prevalent global multidrug-resistant microorganisms globally. Their dissemination is a global concern, particularly by food-producing animals for both animals and humans. The aim of this study was to identify the species and investigate the antibiotic resistance and virulence profile of *Enterococcus* in bovine colostrum. Out of 88 presumptive *Enterococcus* isolates, species identification and susceptibility to 14 antimicrobials were tested using the disk diffusion method. An analysis of the antibiotic resistance and virulence genes was performed on the most prevalent species, using specific PCR assays. *Enterococcus faecalis* (54.5%), *E. faecium* (14.8%) and *E. gallinarum* (6.8%) were the identified species. To the best of our knowledge, this is the first report of *E. gallinarum* in bovine colostrum. The majority of the isolates showed resistance to quinupristin-dalfopristin (95.9%), erythromycin (80.7%), tetracycline (80.7%) and streptomycin (58%). Ninety-two percent of isolates were classified as multidrug-resistant. The most frequently detected resistance genes were *tet*(K) (61.1%), *tet*(M) (75.9%), *tet*(L) (90.7%), *erm*(B) (55.6%) and *ant*(6)-Ia (46.3%). The most prevalent virulence factors were *cpd*, *esp*, *agg* and *cyl*L_L_. *Enterococcus faecium* showed a higher probability of carrying the *erm*(C), *tet*(M), *ace* and *gel*(E) genes (*p* < 0.05). These results demonstrated that colostrum can constitute an important reservoir and vehicle for the dissemination of antibiotic resistance and virulence genes to the three niches included in a One Health perspective (humans, animals and the environment), highlighting the importance of hygiene sanitary measures to mitigate colostrum microbial contamination.

## 1. Introduction

Agriculture and livestock are responsible for more than half of the annual consumption of antibiotics, greatly exceeding hospital consumption [1], which contributes to the development of antibiotic-resistant bacteria in intensive animal production [2,3]. These bacteria can increase the incidence of infectious diseases that are difficult to treat and, consequently, animal mortality, which leads to productivity losses in the sector [4,5]. These pathogens also pose a threat to public health when they colonize the gastrointestinal tracts of young animals and are transmitted to humans as foodborne contaminants, a problem that has been aggravated by the globalization of the food industry and the international trade of live animals [2,4].

Bovine colostrum is a liquid secreted in the first 3–5 days after calving and administered to newborn calves [6,7]. It is characterized by a unique composition, with immunological, nutritional and growth functions, and is extremely important for the health and development of animals [7,8]. A wide variety of bacterial species have been described in colostrum, colonizing the gastrointestinal tract of animals early in life [9,10]. Among these are *Enterococcus*, a bacterial genus that was initially seen as harmless but that is currently considered one of the most important agents of nosocomial infections. In particular, the species *E. faecalis* and *E. faecium*, cause bacteremia, urinary tract infections and endocarditis [11].

Enterococci exhibit an intrinsic resistance to numerous antimicrobial classes, as well as the ability to acquire resistance to many others [12,13]. Acquired resistance by enterococci to vancomycin is one of the most relevant. It is considered that the livestock industry, due to the use of avoparcin (an antibiotic analogous to vancomycin) as a food additive, played an important role in the emergence and dissemination of vancomycin resistance outside the hospital environment [14,15]. Thus, it is hypothesized that colostrum may be responsible for the colonization of calves by *Enterococcus* spp. resistant to antibiotics, contributing to the spread of bacteria carrying antibiotic-resistant genes.

The aim of this study was to identify the species found in colostrum and investigate the antibiotic resistance and virulence profile of *Enterococcus* from colostrum to understand whether bovine colostrum can act as a reservoir and vehicle for the dissemination of antibiotic-resistant *Enterococcus* spp. for animals, humans and the environment.

## 2. Materials and Methods

### 2.1. Isolates

A total of 88 *Enterococcus* spp. isolates were previously obtained from 29 bovine colostrum samples of the Holstein Friesian and Angus breeds. These were collected from 13 dairy farms during the first milking after calving in the Portuguese region of Entre Douro e Minho between December 2019 and January 2021 (Table S1). All procedures and methods were carried out in accordance with the approved guidelines by the Portuguese Veterinary Authority of the Ministry for Agriculture, Sea, Environment and Spatial Planning (Decree law No. 113/2013 of 7 August 2013), for which the current European Communities Council Directive of September 2010 (2010/63/UE) is present.

Ten microliters of each sample was inoculated in selective media for the growth and isolation of *Enterococcus* spp., such as Slanetz–Bartley Agar (Liofilchem^®^ s.r.l., Roseto d. Abruzzi, Italy) at 37 °C for 24–48 h. After the period of incubation, when compatible *Enterococcus* growth was observed in each plaque, 1 to 4 isolates were collected and incubated/isolated in Kanamycin Aesculin Azide Agar (Liofilchem^®^ s.r.l., Roseto d. Abruzzi, Italy). The identification of the isolates was confirmed by routine biochemical methods such as Gram staining, catalase test and growth in the presence of 6.5% NaCl. Subsequently, these were stored in Medical Microbiology Laboratory of the University of Trás-os-Montes and Alto Douro at −20 °C until further analyses.

### 2.2. DNA Extraction

For DNA extraction, the GRS Genomic DNA Kit—Bacteria (GRiSP Research Solutions, Porto, Portugal) was used, according to the manufacturer’s instructions. The concentration and purity of the extracted DNA were evaluated using the ND-100 Spectrophotometer, NanoDrop^®^. In addition, DNA integrity analysis was performed by an agarose gel 1.2%, using the BioRAD’s ChemiDoc™ XRS equipment and BioRAD’s Image Lab^TM^.

### 2.3. Species Identification

*Enterococcus* species identification of the 88 isolates was performed by PCR assay, using the ProFlex^TM^ PCR System thermal cycler (Applied Biosystems, Waltham, MA, USA). Specific primers were tested for 6 possible species: *E. faecalis* (*ddl _E. faecalis_*), *E. faecium* (*ddl _E. faecium_*), *E. gallinarum* (*vanC1*), *E. casseliflavus/E. falavescens* (*vanC2/vanC3*) and *E. durans* (*mur2*). The primer sequences, PCR reaction conditions and amplicon size are shown in Table S2 [16,17]. The followed protocol for DNA amplification was used: a final volume of 25 µL contained 17.67 µL of ultra-pure water, 2.5 µL complete buffer (Bioron, Römerberg, Germany), 0.38 µL of 100 mM MgCl_2_, 0.5 µL of 10 mM deoxynucleotides triphosphate, 0.4 µL of 50 µM of forward primer, 0.4 µL of 50 µM of primer reverse, 0.15 µL of U/µL DFS-Taq DNA polymerase (BIORON^®^, Römerberg, Germany) and 3 µL of DNA (10 ng) sample. Positive and negative controls used in all the experiments belonged to the strain collection of the University of Trás-os-Montes e Alto Douro.

### 2.4. Phenotypic Characterization

The antimicrobial susceptibility test of all isolates (*n* = 88) was performed by the disk diffusion method or the Kirby–Bauer method in Mueller-Hinton II agar (Oxoid^®^, Basingstoke, UK). A total of 14 antibiotics (Liofilchem^®^ s.r.l., Italy) were tested: quinupristin-dalfopristin (15 μg), tetracycline (30 μg), erythromycin (15 μg), streptomycin (300 μg), rifampicin (5 μg), chloramphenicol (30 μg), ciprofloxacin (5 μg), vancomycin (30 μg), linezolid (30 μg), fosfomycin (200 μg), nitrofurantoin (300 μg), teicoplanin (30 μg), ampicillin (10 μg) and gentamicin (120 μg). The concentrations used in each antibiotic complied with the indications of the Clinical and Laboratory Standards Institute (CLSI) [18]. The diameter of the zone of inhibition formed around each disc was measured, and the isolates were classified as sensitive and resistant (intermediate zone of inhibition was classified as resistant) for each antibiotic, according to CLSI recommendations [18].

### 2.5. Genotypic Characterization

The presence of antibiotic resistance genes and virulence factors was investigated in 54 representative isolates belonging to the species *E. faecium* and *E. faecalis*, based on the farm origin and observation of different macroscopic morphologies of colonies. The PCR protocol was performed as described for species identification. The primer sequences, PCR reaction conditions and amplicon size are shown in Tables S3 and S4 [16,19,20,21,22,23,24,25,26].

#### 2.5.1. Detection of Antibiotic Resistance Genes

Fifteen primers were tested for genes encoding resistance to 6 different antibiotics: erythromycin (*erm*(A), *erm*(B), *erm*(C) and *erm*(T)), tetracycline (*tet*(K), *tet*(L), *tet*(M) and *tet*(O)), gentamicin (*aac*(6′)-*aph*(2″)), chloramphenicol (*cat*(A)), quinupristin-dalfopristin (*vat*(D) and *vat*(E)), vancomycin (*van*(A) and *van*(B)) and streptomycin (*ant*(6)-Ia).

#### 2.5.2. Detection of Virulence Genes

Ten specific primers were used for testing genes that encode or are involved in the expression of the following 6 virulence factors: genes encoding collagen-binding protein (*ace*); genes encoding aggregation substance (*agg*); genes encoding sex pheromones (*cpd*); genes encoding gelatinase (*gel*(E)); genes involved in regulating expression of the genes encoding gelatinase (*fsr*); genes encoding enterococcal surface protein (*esp*); and genes encoding cytolysin (*cyl*A, *cyl*B, *cyl*L_L_ and *cyl*M).

### 2.6. Statistical Analysis

Statistical analysis was strictly descriptive via the chi-square (*χ*^2^) independence test and Fisher’s exact test, using the SPSS 15^®^ program (SPSS Inc., Chicago, IL, USA). A probability level lower than 0.05 (*p* < 0.05) was considered statistically significant in the association of variables. It was performed based on the association of *Enterococcus* species with the detected resistance and virulence genes.

## 3. Results

### 3.1. Identification of Enterococcus Species

Three different species were identified in 67 of the isolates: 48 were identified as *E. faecalis*, 13 as *E. faecium* and 6 as *E. gallinarum* (Table 1). No identification of *Enterococcus* species was obtained in 21 of the isolates, which were classified as *Enterococcus* spp.

### 3.2. Phenotypic Characterization

Of the 88 isolates, the majority showed resistance to the following antibiotics: quinupristin-dalfopristin, tetracycline, erythromycin and streptomycin. This was followed by rifampicin, chloramphenicol and ciprofloxacin. Resistance to the remaining antibiotics was below 10%. None of the isolates showed resistance to ampicillin or gentamicin (Table 2).

In total, 39 different antibiotic-resistant phenotypes were found (Table S5), with all isolates showing resistance to at least one antibiotic. Only 7 isolates (8%) exhibited resistance to fewer than three different antimicrobial classes, while the remaining 81 isolates (92%) displayed resistance to three or more different antimicrobial classes, demonstrating a multidrug-resistant profile (Table 2).

### 3.3. Genotypic Characterization

#### 3.3.1. Detection of Antibiotic-Resistant Genes

Among the 54 selected enterococcal isolates of the most prevalent species identified, the most frequently detected antibiotic-resistant genes were three tetracycline resistance genes, including *tet*(K), *tet*(M) and *tet*(L); one erythromycin resistance gene, *erm*(B); and the gene encoding streptomycin resistance, *ant*(6)-Ia. No resistance genes for vancomycin (*van*(A) and *van*(B)) were found (Table 3).

A comparison between the obtained phenotypic and genotypic resistance profiles showed that several isolates (*n* = 30) that did not display phenotypic resistance to a particular antibiotic were found to carry genes that encode resistance to that antibiotic upon analysis using the PCR assay (Table 4 and Table S6).

#### 3.3.2. Detection of Virulence Genes

The most identified virulence factors were *cpd*, *esp*, *agg*, *cyl*L_L_ and *ace* (Table 5). *E. feacalis* was shown to have a greater range of virulence factors than *E. faecium*.

Only one genotype was detected for the presence of the *gel*(E) gene and its regulator, the *fsr* locus (Table 5). Fourteen (25.9%) isolates, eight *E. faecalis* and six *E. faecium*, carried the *gel*(E) gene but did not show the *fsr* locus, resulting in the *gel*(E)^+^*fsr*^−^ genotype.

Furthermore, five different genotypes were identified for the tested *cyl* operon genes (Table 6). Out of the 54 isolates, only 4 (7.4%), all belonging to *E. faecalis*, presented all the four tested genes. In contrast, only one gene (*cyl*L_L_) was identified in *E. faecium*.

**Figure 1:**
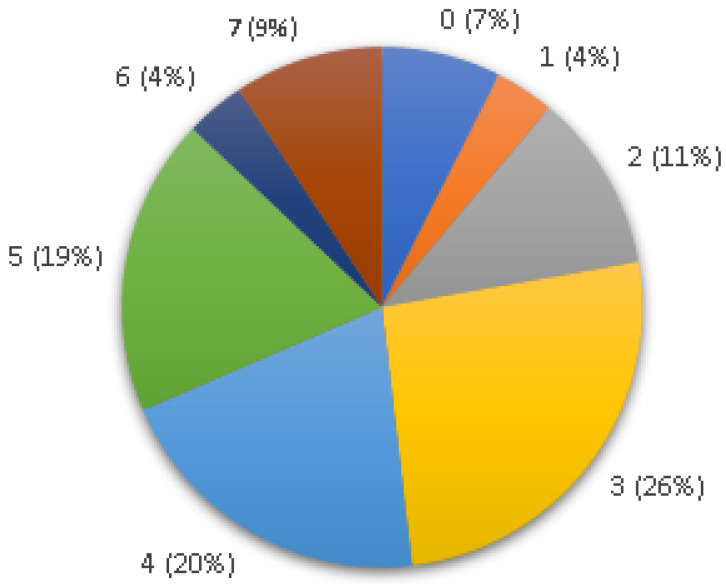
Number of virulence genes identified per *Enterococcus* isolate in this study.

Of the 54 analyzed enterococci isolates, only 4 (7%) did not show amplification for any virulence factor. The remaining isolates were identified with two (*n* = 6; 11%), three (*n* = 14; 26%), four (*n* = 11; 20%), five (*n* = 10; 19%) or more (*n* = 7; 13%) virulence factors (Figure 1).

The statistical analysis showed significant results (*p* < 0.05) for the resistance genes including *erm*(C) and *tet*(M), with *E. faecium* having a greater probability of having both genes. For the virulence genes, significant results were observed for the *ace* and *gel*(E) genes, which were also more prevalent in *E. faecium* (Table 7).

## 4. Discussion

Bovine colostrum is a rich feed that transfers passive immunity to calves. Moreover, the microbiological quality of colostrum is considered one of the key factors of successful calf management and health [6]. *E. faecalis* and *E. faecium* were the *Enterococcus* species predominantly isolated from colostrum, which was consistent with previous findings for bovine and human colostrum [9,27]. Our obtained results are in concordance with these findings, although a small percentage of the isolates were classified as *E. gallinarum.* To the best of our knowledge, this is the first report of their presence in bovine colostrum; however, they have already been reported in raw cow’s milk [28]. Unfortunately, the species was not identified in 21 isolates, making it necessary to extend the analysis with specific primers for other species.

There are a variety of reasons for microbial contamination in colostrum. The presence of enterococci in colostrum may originate from mammary gland infection or be indicative of inadequate hygienic and sanitary conditions during milking, such as the fecal contamination of the animal’s skin, milking instruments or workers’ hands [27,29]. Some of the samples had been previously pasteurized, indicating that the bacteria were not eliminated due to inadequate heat treatment or that the colostrum may have suffered subsequent contamination [30]. These reasons may explain why, in our study, four analyzed samples that were also pasteurized (Table S1) showed enterococci growth.

Most of the isolates under study showed antibiotic resistance to quinupristin-dalfopristin (95.5%), as expected, since *Enterococcus* isolates are intrinsically resistant to quinupristin-dalfopristin [18]. However, a slightly lower percentage (60.8%) of *Enterococcus* spp. resistant to quinupristin-dalfopristin was detected in mastitis cow milk [31]. Tetracycline and erythromycin also showed high levels of resistance (80.7%), in contrast to what was observed in *E. faecalis* and *E. faecium* obtained from cheeses [32]. A little more than half (58.0%) of the isolates showed resistance to streptomycin, in contrast to what was observed in dairy products, processed meats and chicken carcasses [33].

Less than half of the isolates were resistant to rifampicin (47.8%), chloramphenicol (26.1%) and ciprofloxacin (11.4%). In obtained *E. faecalis* and *E. faecium* isolates from cheese and chicken, a high resistance to rifampicin and ciprofloxacin and a low resistance to chloramphenicol was reported [32,34]. Less than 10% of the colostrum isolates showed resistance to antimicrobials such as linezolid, fosfomycin and nitrofurantoin. Similar results have been reported from ready-to-eat dairy products in isolates of *E. faecium*, *E. faecalis*, *E. gallinarum* and *E. casseliflavius* [35]. None of the isolates showed resistance to ampicillin or gentamicin, in contrast to what was observed in *E. faecalis* and *E. faecium* isolated from raw cow’s milk [36].

One of the most important resistances to evaluate in *Enterococcus* spp. is resistance to glycopeptides (vancomycin and teicoplanin). In this study, the percentage of resistance was low. Studies conducted on raw cow’s milk with *E. faecium* and *E. faecalis* revealed a higher resistance to both antibiotics, in which 37% were resistant to vancomycin and 44% were resistant to teicoplanin [36].

The species that showed resistance to a greater number of antibiotics in the phenotypic analysis was *E. faecalis* with a total of 11 antibiotics, followed by *E. gallinarum* with 8 antibiotics and, finally, *E. faecium* with 7 antibiotics. Several studies have also reported that *E. faecalis* is the species with the most resistance to the greatest number of antibiotics [33,37]. All isolates under study showed resistance to at least one antimicrobial class, and a very high percentage (92%) of isolates were classified as multidrug-resistant. In agreement with these findings, another study described the presence of multidrug-resistant *E. faecalis* and *E. faecium* isolates in chickens, fresh and fermented meat, raw and fermented milk, and cheese [34].

From the genotypic characterization, *tet*(L) was the most detected of the screened tetracycline-resistant genes. While in another study, the most frequently detected gene was *tet*(M) [35]. In the case of erythromycin resistance, the *erm*(B) gene has been identified as the most prevalent worldwide [37], which is in line with the results obtained in our study. The *ant*(6)-Ia gene was detected in half of the isolates that showed phenotypic resistance to streptomycin. Similarly, samples of game meat, from which five isolates of *E. faecium* and *E. faecalis* are resistant to this antibiotic, did not show this gene [38].

The *van*(A) and *van*(B) genes, individually or in combination, are the most commonly found genotypes among *Enterococcus* spp. with acquired resistance mechanisms to vancomycin in humans and animals [39]. Although one of the isolates of *E. faecalis*, from bovine colostrum, was phenotypically resistant to vancomycin, the *van*(A) and *van*(B) genes were not amplified, which agrees with results obtained in studies of human colostrum and milk [40].

Several isolates did not show phenotypic resistance to certain antibiotics (tetracycline, streptomycin, chloramphenicol, erythromycin and gentamicin), but genes that encode resistance to corresponding antibiotics were detected. This can be explained by the negative regulation of the resistance gene, low levels of gene expression or inactive gene product expression. Since environmental factors can interfere with gene expression, this may happen when an isolate that is not resistant to an antibiotic in vitro, under conditions found in humans or animals, proves to be resistant [24].

The presence of antibiotic-resistant genes does not make a bacterium pathogenic; to be able to colonize and subsequently cause disease, it requires the presence and expression of several virulence factors. The *cpd* and *esp* virulence factors were the most detected in *E. faecalis* and *E. faecium* colostrum isolates, as previously observed in ready-to-eat shrimp [41]. Alternatively, the *agg* and *ace* genes were found in lower proportions in *E. faecalis* isolates from dairy and meat products than in bovine colostrum isolates [33].

The *gel*(E) gene was detected in isolates from this study; however, its regulator, *fsr* locus, was not detected in any of the isolates. Previous studies observed that isolates with gelatinase activity present amplification for *gel*(E) and its regulator *fsr* [42,43]. According to these data, the isolates of this study do not present gelatinase activity (*gel*(E)^+^ *fsr*^−^ genotype) since, even if they have the gene, it may be silenced [32]. In our study, the presence of the four tested *cyl* operon genes were observed in four isolates. The expression of this bacteriocin requires the presence of the entire *cyl* operon, which means that only four of the studied isolates (all *E. faecalis*) have the possibility of expressing it [44].

*E. feacalis* has been shown to have a greater range of virulence factors than *E. faecium*. *E. faecium* was more likely to carry the *ace* and *gel*(E) virulence genes than *E. faecalis*. However, other studies have reported that the prevalence of these virulence genes and others (*esp* and *agg*) was significantly higher in *E. faecalis* isolates [45,46]. Only 4 of the 54 analyzed *Enterococcus* showed no virulence factors. The number of virulence factors in obtained enterococci from bovine colostrum contradicts the hypothesis that these determinants are more prevalent in clinical isolates than in isolates recovered from food, food-producing animals or wild animals [42,47,48].

## 5. Conclusions

The present study demonstrated that bovine colostrum can be a reservoir for *Enterococcus* spp. resistant to antibiotics, which possess significant pathogenic potential. As colostrum is an essential source of immunity and nutrition for calves, when present, these multidrug-resistant bacteria are likely to be transmitted to them. Consequently, it is likely that these microorganisms persist in the food or products derived from these calves, such as milk or their carcasses, that enter the human food chain. This cycle of dissemination must be stopped as it culminates in a food safety and public health problem. In order to prevent colostrum from being a reservoir and vehicle for the spread of antibiotic-resistant bacteria, it is important to make a more prudent use of antimicrobials and to apply good hygiene and manufacturing practices during the collection and management of colostrum.

## Figures and Tables

**Table 1:** *Enterococcus* species identified from bovine colostrum in this study.

Species	No.	%
*E. faecalis*	48	54.5
*E. faecium*	13	14.8
*E. gallinarum*	6	6.8
*E. casseliflavus*/*E. flavescens*	0	0.0
*E. durans*	0	0.0
*Enterococcus* spp.	21	23.9
Total	88	100

No.: Number of isolates; %: percentage of isolates.

**Table 2:** Phenotypic profile of antibiotic-resistant *Enterococcus* isolates (*n* = 88) obtained from bovine colostrum by the disk diffusion method performed in this study.

Antibiotics	Species								Total	
	*E. faecalis*		*E. faecium*		*E. gallinarum*		*Enterococcus* spp.			
	No.	%	No.	%	No.	%	No.	%	No.	%
Ampicillin ^1^	0	0.0	0	0.0	0	0.0	0	0.0	0	0.0
Vancomycin ^2^	2	4.2	0	0.0	0	0.0	3	14.3	5	5.7
Teicoplanin ^2^	0	0.0	0	0.0	0	0.0	1	4.8	1	1.1
Fosfomycin ^3^	3	6.3	0	0.0	0	0.0	0	0.0	3	3.4
Ciprofloxacin ^4^	5	10.4	1	7.7	1	16.7	3	14.3	10	11.4
Rifampicin ^5^	25	52.1	4	30.8	4	66.7	9	42.9	42	47.7
Erythromycin ^6^	40	83.3	9	69.2	5	83.3	17	81	71	80.7
Tetracycline ^7^	39	81.3	11	84.6	5	83.3	16	76.2	71	80.7
Nitrofurantoin ^8^	2	4.2	0	0.0	0	0.0	0	0.0	2	2.3
Chloramphenicol ^9^	15	31.3	2	15.4	2	33.3	4	19.1	23	26.1
Quinupristin-dalfopristin ^10^	48	100	10	76.9	5	83.3	21	100	84	95.5
Linezolid ^11^	2	4.2	0	0	1	16.7	1	4.8	4	4.6
Gentamicin ^12^	0	0	0	0	0	0	0	0	0	0
Streptomycin ^12^	24	50.0	8	61.5	4	66.7	15	71.4	51	58.0

No.: Number of isolates; %: percentage of isolates. Antimicrobial classes: ^1^: penicillins; ^2^: glycopeptides; ^3^: fosfoycins; ^4^: fluoroquinolones; ^5^: ansamycins; ^6^: macrolides; ^7^: tetracyclines; ^8^: nitrofurantoins; ^9^: phenicols; ^10^: streptogramins; ^11^: oxazolidinones; ^12^: aminoglicosides.

**Table 3:** Antibiotic-resistant genes in *E. faecalis* and *E. faecium* obtained from bovine colostrum in this study (*n* = 54).

Resistance Genes	Species				Total	
	*E. faecalis*		*E. faecium*			
	No.	%	No.	%	No.	%
*erm*(A)	0	0.0	0	0.0	0	0.0
*erm*(B)	26	60.5	4	36.4	30	55.6
*erm*(C)	4	9.3	6	54.6	10	18.6
*tet*(K)	27	62.8	6	54.6	33	61.1
*tet*(M)	30	69.8	11	100	41	75.9
*tet*(L)	40	93.0	9	81.8	49	90.7
*tet*(O)	2	4.7	0	0.0	2	3.7
*aac*(6′)-*aph*(2″)	2	4.7	0	0.0	2	3.7
*cat*(A)	9	20.9	5	45.5	14	25.9
*vat*(D)	0	0	0	0.0	0	0.0
*vat*(E)	1	2.3	0	0.0	1	1.9
*van*(A)	0	0.0	0	0.0	0	0.0
*van*(B)	0	0.0	0	0.0	0	0.0
*ant*(6)-Ia	19	44.2	6	54.6	25	46.3

No.: Number of isolates; %: percentage of isolates.

**Table 4:** Antibiotic resistance genes identified in enterococci isolates that did not exhibit the corresponding resistance phenotype.

Species	Phenotypical Resistance	No.	Resistance Genes	No.
*E. faecalis*	Not resistant to tetracycline	9	*tet*(L)	3
			*tet*(K)	1
			*tet*(K)*-tet*(L)	3
	Not resistant to chloramphenicol	32	*cat*(A)	3
	Not resistant to gentamicin	43	*aac*(6′)-*aph*(2″)	2
	Not resistant to streptomycin	21	*ant*(6)-la	9
*E. faecium*	Not resistant to eritromycin	4	*erm*(C)	2
	Not resistant to tetracycline	1	*tet*(K)-*tet*(M)-*tet*(L)	1
	Not resistant to chloramphenicol	10	*cat*(A)	6

No.: Number of isolates.

**Table 5:** Virulence factors detected in *E. feacalis* (*n* = 43) and *E. faecium* (*n* = 11) isolated from bovine colostrum.

Virulence Genes	*E. faecalis*		*E. faecium*		Total	
	No.	%	No.	%	No.	%
*esp*	29	67.4	8	72.7	37	68.5
*ace*	15	34.9	9	81.8	24	44.4
*cpd*	35	81.4	9	81.8	44	81.5
*agg*	26	60.5	7	63.6	33	61.1
*gel*(E)	8	18.6	6	54.6	14	25.9
*fsr*	0	0.0	0	0.0	0	0.0
*cyl*A	7	16.3	0	0.0	7	13.0
*cyl*B	9	20.9	0	0.0	9	16.7
*cyl*M	4	9.3	0	0.0	4	7.4
*cyl*L_L_	21	48.8	4	36.4	25	46.3

No.: Number of isolates; %: percentage of isolates.

**Table 6:** Detection of *cyl* operon isolated in *E. faecalis* and *E. faecium* analyzed in this study.

Operon *cyl*	*E. faecalis*		*E. faecium*		Total	
	No.	%	No.	%	No.	%
*cyl*L_L_	14	32.6	3	27.3	17	31.5
*cyl*B	1	2.3	0	0.0	1	1.9
*cyl*B*-cyl*L_L_	1	2.33	0	0.0	1	1.9
*cyl*A*-cyl*B*-cyl*L_L_	3	7.0	0	0.0	3	5.6
*cyl*A*-cyl*B*-cyl*M*-cyl*L_L_	4	9.3	0	0.0	4	7.4

No.: Number of isolates; %: percentage of isolates.

**Table 7:** Statistical results based on the detected *Enterococcus* species in relation to the resistance and virulence genes analyzed in this study.

Gene		*E. faecalis*	*E. faecium*	*χ* ^2^	*p*
		No.	No.		
*erm*(C)	Positive	39	5	11.882	0.003
	Negative	4	6		
*tet*(M)	Positive	13	0	4.380	0.048
	Negative	30	11		
*ace*	Positive	28	2	7.815	0.007
	Negative	15	9		
*gel*(E)	Positive	35	5	5.892	0.024
	Negative	8	6		

No.: Number of isolates; *χ*^2^: chi-square; *p*: probability.

## Data Availability

Not applicable.

## Supplementary Materials

The following supporting information can be downloaded at https://www.mdpi.com/article/10.3390/ani13121900/s1. Table S1: Characteristics of the 29 colostrum samples from 13 dairy farms used in this study, as well as the number of *Enterococcus* isolates obtained in each farm; Table S2: Nucleotide sequence of the primers used in the identification of *Enterococccus* species, as well as amplification conditions and size of the band obtained; Table S3: Nucleotide sequence of primers for amplification of antibiotic-resistant genes in *Enterococcus* spp., as well as amplification conditions and size of the band obtained; Table S4: Nucleotide sequence of primers for amplification of virulence factors in *Enterococcus* spp., as well as amplification conditions and size of the band obtained; Table S5: Resistance phenotypes obtained in *E. faecalis*, *E. faecium*, *E. gallinarum* and *Enterococcus* spp. from bovine colostrum (*n* = 88); Table S6: Phenotype and genotype characterization of *E. faecalis* (*n* = 43) and *E. faecium* (*n* = 11) isolates.
