# Supplementary material for: Analysis of Antibiotic-Resistant and Virulence Genes of Enterococcus Detected in Calf Colostrum—One Health Perspective
Source: Animals (Basel). 2023 Jun 7;13(12):1900. doi: 10.3390/ani13121900 (PMC10295430; doi:10.3390/ani13121900)
Supplement: Supplementary file 1 [file animals-13-01900-s001.zip › animals-2424335-supplementary.pdf]

animals

MDPI

Supplementary Materials

Analysis of antibiotic-resistant and virulence genes of *Enterococcus* detected in calf colostrum – One Health perspective

Sandra Cunha [1,†], Carla Miranda [1-3,†,*], Ângela Martins [4,5,6], Rúben Soares [1], Manuel Maia [1], Filipe Silva [5,6,7], Gilberto Igrejas [2,8,9] and Patrícia Poeta [1,2,5,6]

1 Microbiology and Antibiotic Resistance Team (MicroART), Department of Veterinary Sciences, University of Trás-os-Montes and Alto Douro, 5000-801 Vila Real, Portugal; al62192@utad.eu; carlisabelmi@utad.pt; al70477@alunos.utad.pt; al60199@alunos.utad.pt; ppoeta@utad.pt
2 Associated Laboratory for Green Chemistry (LAQV-REQUIMTE), University NOVA of Lisbon, 1099-085 Caparica, Portugal;
3 Toxicology Research Unit (TOXRUN), University Institute of Health Sciences, Advanced Polytechnic and University Cooperative (IUCS-CESPU), CRL, 4585-116 Gandra, Portugal;
4 Department of Zootechnics, University of Trás-os-Montes and Alto Douro, Vila Real, Portugal; angela@utad.pt
5 Veterinary and Animal Research Centre (CECAV), University of Trás-os-Montes e Alto Douro, 5000-801 Vila Real, Portugal;
6 Associate Laboratory for Animal and Veterinary Science (AL4AnimalS), University of Trás-os-Montes and Alto Douro, 5000-801 Vila Real, Portugal;
7 Department of Veterinary Sciences, University of Trás-os-Montes and Alto Douro, 5000-801 Vila Real, Portugal; fsilva@utad.pt
8 Department of Genetics and Biotechnology, University of Trás-os-Montes and Alto Douro, 5000-801 Vila Real, Portugal; gigrejas@utad.pt
9 Functional Genomics and Proteomics Unit, University of Trás-os-Montes and Alto Douro, 5000-801 Vila Real, Portugal
† These authors contributed equally to this work.
* Correspondence: carlisabelmi@utad.pt

Citation: Cunha, S.; Miranda, C.; Martins, Â.; Soares, R.; Maia, M.; Silva, F.; Igrejas, G.; Poeta, P. Analysis of Antibiotic-Resistant and Virulence Genes of *Enterococcus* Detected in Calf Colostrum—One Health Perspective. *Animals* **2023**, *13*, x. https://doi.org/10.3390/xxxxx

Academic Editor: Amit Vikram

Received: 15 May 2023
Revised: 2 June 2023
Accepted: 5 June 2023
Published: date

Copyright: © 2023 by the authors. Submitted for possible open access publication under the terms and conditions of the Creative Commons Attribution (CC BY) license (https://creativecommons.org/licenses/by/4.0/).

Table S1. Characteristics of the 29 colostrum samples from 13 dairy farms used in this study, as well as the number of *Enterococcus* isolates obtained in each farm.

Sample	No. of isolates	Exploration	Localization	Year of collection	Conservation method	Pasteurization
1	3	A	Vila do Conde	2019	Refrigeration	No
5	4	B	Vila do Conde	2019	Freezing	Yes
6	1	B	Vila do Conde	2019	Fresh	No
7	4	A	Vila do Conde	2020	Refrigeration	No
9	4	C	Póvoa de Varzim	2019	Fresh	No
10	4	D	Maia	2020	Fresh	No
11	4	E	Maia	2020	Fresh	No
12	4	F	Vila do Conde	2020	Fresh	No
13	2	F	Vila do Conde	2020	Fresh	No
14	4	D	Maia	2020	Fresh	No
15	4	G	Vila do Conde	2020	Fresh	No
16	4	H	Vila do Conde	2020	Fresh	No
18	3	A	Vila do Conde	2020	Refrigeration	No
21	2	H	Vila do Conde	2020	Fresh	No
22	2	E	Maia	2020	Fresh	No
25	3	I	Vila do Conde	2020	Fresh	No
26	4	J	Vila do Conde	2020	Fresh	No
27	2	J	Vila do Conde	2020	Fresh	No
28	4	J	Vila do Conde	2020	Fresh	No
30	2	L	Vila do Conde	2020	Fresh	No
31	4	C	Póvoa de Varzim	2020	Fresh	No
32	4	M	Vila do Conde	2020	Fresh	No
34	2	M	Vila do Conde	2020	Fresh	No
35	1	G	Vila do Conde	2020	Fresh	No
36	4	G	Vila do Conde	2020	Fresh	No
37	2	N	Vila do Conde	2021	Refrigeration	Yes
38	1	N	Vila do Conde	2021	Refrigeration	Yes
39	2	N	Vila do Conde	2021	Refrigeration	Yes
40	4	C	Póvoa de Varzim	2021	Fresh	No

No. number of isolates.

Table S2. Nucleotide sequence of the primers used in the identification of *Enterococccus* species, as well as amplification conditions and size of the band obtained.

Gene	**Species**	**Primer sequence (5′→3′)**	**Amplification conditions**	**Size (pb)**	**Reference**
ddl	*E. faecalis*	ATCAAGTACAGTTAGTCT<br>ACGATTCAAAGCTAACTG	94ºC, 2min (1cycle)<br>94ºC, 1min<br>54ºC, 1min (30cycles)<br>72ºC, 1min<br>72ºC, 10min (1cycle)	941	[16]
ddl	*E. faecium*	TAGAGACATTGAATATGCC<br>TCGAATGTGCTACAATC	94ºC, 2min (1cycle)<br>94ºC, 1min<br>54ºC, 1min (30cycles)<br>72ºC, 1min<br>72ºC, 10min (1cycle)	550	[16]
vanC1	*E. gallinarum*	GGTATCAAGGAAACCTC<br>CTTCCGCCATCATAGCT	94ºC, 2min (1cycle)<br>94ºC, 30s<br>58ºC, 2min (40cycles)<br>72ºC, 2min<br>72ºC, 6min (1cycle)	882	[16]
vanC2, vanC3	*E. casseliflavus*<br>*E. flavescens*	CTCCTACGATTCTCTTG<br>CGAGCAAGACCTTTAAG	94ºC, 3min (1cycle)<br>94ºC, 1min<br>58ºC, 1min (30cycles)<br>72ºC, 1min<br>72ºC, 10min (1cycle)	439	[16]
mur2	*E. durans*	CGTCAGTACCCTTCTTTTGCAG AGTC<br>GCATTATTACCAGTGTTAGTGG TTG	94ºC, 2min (1cycle)<br>94ºC, 1min<br>60ºC, 15min (30cycles)<br>72ºC, 1.5min<br>72ºC, 7min (1cycle)	521	[17]

Pb: base pairs; °C: degree Celsius; min: minutes; s: seconds.

Table S3. Nucleotide sequence of primers for amplification of antibiotic-resistant genes in *Enterococcus* spp., as well as amplification conditions and size of the band obtained.

Gene	**Primer sequence (5′→3′)**	**Amplification conditions**	**Size (pb)**	**Reference**
erm(A)	TCTAAAAAGCATGTAAAAGAA<br>CTTCGATAGTTTATTAATATTAGT	93^{0}C, 3min (1cycle)<br>93^{0}C, 1min<br>52^{0}C, 1min (35cycles)<br>72^{0}C, 1min<br>72^{0}C, 5min (1cycle)	645	[19]
erm(B)	GAAAAGGRACTCAACCAAATA<br>AGTAACGGTACTTAAATTGTTTAC	93^{0}C, 3min (1cycle)<br>93^{0}C, 1min<br>52^{0}C, 1min (35cycles)<br>72^{0}C, 1min<br>72^{0}C, 5min (1cycle)	639	[19]
erm(C)	TCAAAACATAATATAGATAAA<br>GCTAATATTGTTTAAATCGTCAAT	93^{0}C, 3min (1cycle)<br>93^{0}C,1min<br>52^{0}C, 1min (35cycles)<br>72^{0}C, 2min<br>72^{0}C, 5min (1cycle)	642	[19]
tet(K)	TTAGGTGAAGGGTTAGGTCC<br>GCAAACTCATTCCAGAAGCA	94^{0}C, 1min (1cycle)<br>94^{0}C, 1min<br>55^{0}C, 2min (30cycles)<br>72^{0}C, 2min<br>72^{0}C, 10min (1cycle)	697	[20]
tet(M)	GTTAAATAGTGTTCTTGGAG<br>CTAAGATATGGCTCTAACAA	94^{0}C, 1min (1cycle)<br>94^{0}C, 1min<br>55^{0}C, 2min (30cycles)<br>72^{0}C, 2min<br>72^{0}C, 10min (1cycle)	576	[20]
tet(L)	CATTTGGTCTTATTGGATCG<br>ATTACACTTCCGATTTCGG	94^{0}C, 1min (1cycle)<br>94^{0}C, 1min<br>50^{0}C, 1min (30cycles)<br>72^{0}C, 1min<br>72^{0}C, 10min (1cycle)	456	[20]
tet(O)	ACGGARAGTTTATTGTATACC<br>TGGCGTATCTATAATGTTGAC	94^{0}C, 5min<br>94^{0}C, 30s<br>60^{0}C, 30s (25cycles)<br>72^{0}C, 30s<br>72^{0}C, 7min	171	[21]

Pb: base pairs; °C: degree Celsius; min: minutes; s: seconds.

Table S3 (Cont.). Nucleotide sequence of primers for amplification of antibiotic-resistant genes in *Enterococcus* spp., as well as amplification conditions and size of the band obtained (continuation).

Gene	**Primer sequence (5′→3′)**	**Amplification conditions**	**Size (pb)**	**Reference**
aac(6′)-*aph*(2′′)	CCAAGAGCAATAAGGGCATA<br>CACTATCATACCACTACCG	94ºC, 3min (1cycle)<br>94ºC, 30s<br>60ºC, 45s (30cycles)<br>72ºC, 2min<br>72ºC, 6min (1cycle)	220	[22]
cat(A)	GGATATGAAATTTATCCCTC<br>CAATCATCTACCCTATGAAT	94ºC, 5min (1cycle)<br>94ºC, 1min<br>50ºC, 1min (30cycles)<br>72ºC, 2,5min<br>72ºC, 7min (1cycle)	486	[20]
vat(E)	ACGTTACCCATCACTATG<br>GCTCCGATAATGGCACCGAC	94ºC, 2min (1cycle)<br>94ºC, 1min<br>55ºC, 2min (40cycles)<br>72ºC, 3min<br>72ºC, 5min (1cycle)	282	[23]
van(A)	GGGAAAACGACAATGC<br>GTACAATGCGGCCGTTA	96ºC, 2min (1cycle)<br>94ºC, 30s<br>50ºC, 30s (35cycles)<br>72ºC, 1min<br>72ºC, 10min (1cycle)	732	[16]
van(B)	ATGGGAAGCCGATAGTC<br>GATTTCGTTCCTCGACC	95ºC, 10min (1cycle)<br>94ºC, 30s<br>58ºC, 30s (30cycles)<br>72ºC, 30s<br>72ºC, 10min (1cycle)	635	[16]
ant(6)-Ia	ACTGGCTTAATCAATTTGGG<br>GCCTTTCCGCCACCTCACCG	94ºC, 10min (1cycle)<br>94ºC, 30s<br>58ºC, 30s (35cycles)<br>72ºC, 30s<br>72ºC, 10min (1cycle)	577	[22]

Pb: base pairs; °C: degree Celsius; min: minutes; s: seconds.

Table S4. Nucleotide sequence of primers for amplification of virulence factors in *Enterococcus* spp., as well as amplification conditions and size of the band obtained.

Gene	**Primer sequence (5′→3′)**	**Amplification conditions**	**Size (pb)**	**Reference**
esp	TTGCTAATGCTAGTCCACGACC<br>GCGTCAACACTTGCATTGCCGAA	95ºC, 2min (1cycle)<br>94ºC, 45s<br>63ºC, 45s (30cycles)<br>72ºC, 1min<br>72ºC, 4min (1cycle)	955	[24]
ace	AAAGTAGAATTAGATCCACAC<br>TCTATCACATTCGGTTGCG	94ºC, 3min (1cycle)<br>94ºC,1min<br>56ºC, 1min (30cycles)<br>72ºC, 1min<br>72ºC, 5min (1cycle)	248	[25]
gel(E)	AGTTCATGTCTATTTTCTTCAC<br>CTTCATTATTTACACGTTTG	94ºC, 3min (1cycle)<br>94ºC, 1min<br>56ºC, 1min (30cycles)<br>72ºC, 1min<br>72ºC, 5min (1cycle)	403	[24]
agg	AAGAAAAAGAAGTAGACCAAC<br>AAACGGCAAGACAAGTAAATA	94ºC, 1min (1cycle)<br>94ºC, 1min<br>55ºC, 2min (30cycles)<br>72ºC, 2min<br>72ºC, 10min (1cycle)	1553	[24]
fsr	ACCAGAATCGACCAATGAAT<br>GCCCCTCATAACTCAATACC	95ºC, 5min (1cycle)<br>94ºC, 30s<br>60ºC,30s (30cycles)<br>72ºC, 2.5min<br>72ºC, 10min (1cycle)	3268	[24]
cpd	TGGTGGGTTATTTTTCAATTC<br>TACGGCTCTGGCTTACTA	95ºC, 2min (1cycle)<br>94ºC, 45s<br>49ºC, 45s (30cycles)<br>72ºC, 1min<br>72ºC, 4min (1cycle)	782	[24]
*cyl*A	TGGATGATAGTGATAGGAAGT<br>TCTACAGTAAATCTTTCGTCA	95ºC, 2min (1cycle)<br>94ºC, 45s<br>49ºC, 45s (35cycles)<br>72ºC, 1min<br>72ºC, 4min (1cycle)	517	[24]
*cyl*B	ATTCCTACCTATGTTCTGTTA<br>AATAAACTCTTCTTTTCCAAC	95ºC, 2min (1cycle)<br>94ºC, 45s<br>49ºC, 45s (35cycles)	843	[24]

		72ºC, 1min<br>72ºC, 4min (1cycle)		
*cyl*M	CTGATGGAAAGAAGATAGTAT<br>TGAGTTGGTCTGATTACATTT	95ºC, 2min (1cycle)<br>94ºC, 45s<br>49ºC, 45s (35cycles)<br>72ºC, 1min<br>72ºC, 4min (1cycle)	742	[24]
*cyl*LL	GATGGAGGGTAAGAATTATGG<br>GCTTCACCTCACTAAGTTTTATAG	94ºC, 3min (1cycle)<br>94ºC, 1min<br>55ºC, 1min (35cycles)<br>72ºC, 2min<br>72ºC, 7min (1cycle)	253	[26]

Pb: base pairs; °C: degree Celsius; min: minutes; s: seconds.

Table S5. Resistance phenotypes obtained in *E. faecalis, E. faecium*, *E. gallinarum* and *Enterococcus* spp. from bovine colostrum (n=88).

Species	Resistance phenotype	No. antimicrobial classes	No.	%
E. faecalis	QD	1	1	2.1
	E-QD	2	1	2.1
	TET-QD	2	2	4.2
	E-QD-RD	3	2	4.2
	TET-QD-RD	3	3	6.3
	TET-E-QD	3	4	8.3
	TET-E-QD-S	4	5	10.4
	TET-E-QD-RD	4	1	2.1
	E-C-QD-S	4	1	2.1
	E-QD-RD-S	4	1	2.1
	TET-E-C-QD	4	3	6.3
	E-C-QD-RD	4	1	2.1
	VA-CIP-QD-RD	4	1	2.1
	TET-E-CIP-QD-FOS	5	1	2.1
	E-C-QD-RD-S	5	1	2.1
	TET-E-QD-RD-S	5	9	18.8
	TET-E-C-QD-S	5	2	4.2
	TET-E-CIP-C-QD	5	1	2.1
	TET-E-C-QD-RD	5	1	2.1
	TET-QD-RD-S-LNZ	5	1	2.1
	TET-E-C-QD-RD-S	6	3	6.3
	TET-E-CIP-QD-F-FOS	6	1	2.1
	TET-E-CIP-C-QD-F-FOS	7	1	2.1

	VA-TET-E-C-QD-RD-S-LNZ	8	1	2.1
E. faecium	E-QD	2	1	7.7
	TET-S	2	1	7.7
	E-QD-RD	3	1	7.7
	TET-QD-S	3	1	7.7
	TET-E-S	3	1	7.7
	TET-E-QD-S	4	2	15.4
	TET-E-QD-RD	4	1	7.7
	TET-QD-RD-S	4	2	15.4
	TET-E-CIP-QD	4	1	7.7
	TET-E-C-QD-S	5	1	7.7
E. gallinarum	TET-QD-RD	3	1	16.7
	TET-E-C-S	4	1	16.7
	TET-E-QD-S	4	1	16.7
	E-C-QD-RD-S	5	1	16.7
	TET-E-QD-RD-S	5	1	16.7
	TET-E-CIP-QD-RD-LNZ	6	1	16.7
Enterococcus spp.	QD	1	1	4.8
	TET-QD-S	3	1	4.8
	E-QD-RD	3	1	4.8
	TET-E-QD-S	4	6	28.6
	TET-E-QD-RD	4	2	9.5
	E-C-QD-S	4	1	4.8
	TET-QD-RD-S	4	1	4.8
	VA-CIP-QD-RD	4	1	4.8
	VA-E-C-QD-S	5	1	4.8
	TET-E-QD-RD-S	5	1	4.8
	TET-E-CIP-QD-S	5	1	4.8
	TET-E-CIP-QD-RD	5	1	4.8
	VA-TET-E-C-QD-S	6	1	4.8
	TET-E-C-QD-RD-S	6	1	4.8
	TEC-TET-E-QD-RD-S	6	1	4.8

AMP - ampicillin; VA - vancomycin; TEC - teicoplanin; TET, - tetracycline; E - erythromycin; CIP - ciprofloxacin; C - chloramphenicol; QD - quinupristin-dalfopristin; F - nitrofurantoin; RD - rifampicin; FOS - fosfomycin; CN - gentamicin; S - streptomycin; LNZ - linezolid; No. - number of isolates; % - percentage of isolates.

Table S6. Phenotype and genotype characterization of *E. faecalis* (n=43) and *E. faecium* (n=11) isolates.

Sample	Isolated	Specie	Resistance Phenotype	Resistance Genotype
1	1	*E. faecalis*	VA-CIP-QD-RD	*tet*(K)-*tet*(L)
1	3	*E. faecalis*	E-QD-RD	*tet*(L)-*vat*(E)
5	1	*E. faecalis*	TET-QD-RD	*tet*(K)-*tet*(M)-*tet*(L)
5	3	*E. faecalis*	TET-E-CIP-QD-FOS	*erm*(B)-*erm*(C)-*tet*(K)-*tet*(M)-*tet*(L)-*ant*(6)-Ia
5	4	*E. faecalis*	TET-QD	*tet*(K)-*tet*(M)-*tet*(L)
7	2	*E. faecalis*	E-C-QD-S	*erm*(B)-*tet*(K)-*tet*(L)-*ant*(6)-Ia
7	4	*E. faecalis*	E-C-QD-RD-S	*erm*(B)-*tet*(L)-*ant*(6)-Ia
9	1	*E. faecalis*	TET-E-QD	*erm*(B)-*erm*(C)-*tet*(M)-*tet*(L)-*ant*(6)-Ia
9	2	*E. faecalis*	TET-E-QD	*erm*(B)-*tet*(K)-*tet*(M)-*tet*(L)-*ant*(6)-Ia
9	3	*E. faecalis*	TET-E-QD-S	*erm*(B)-*tet*(M)-*tet*(L)-*ant*(6)-Ia
9	4	*E. faecalis*	TET-E-QD	*erm*(B)-*tet*(K)-*tet*(M)-*tet*(L)-*ant*(6)-Ia
10	1	*E. faecalis*	TET-QD-RD	*tet*(M)-*tet*(L)
10	2	*E. faecalis*	TET-QD-RD	*tet*(K)-*tet*(M)-*tet*(L)
10	3	*E. faecalis*	TET-E-QD-RD-S	*tet*(M)
11	2	*E. faecium*	TET-S	*tet*(K)-*tet*(M)-*tet*(L)-*ant*(6)-Ia
11	3	*E. faecalis*	TET-E-QD	*erm*(B)-*ant(6)-la*
11	4	*E. faecalis*	TET-QD	*tet*(K)-*tet*(M)-*tet*(L)-*ant*(6)-Ia
12	1	*E. faecium*	TET-E-C-QD-S	*erm*(B)-*tet*(K)-*tet*(M)-*tet*(L)-*ant(6)-la*
12	2	*E. faecalis*	TET-E-C-QD-S	*erm*(B)-*tet*(K)-*tet*(L)-*cat*(A)-*ant(6)-la*
12	3	*E. faecalis*	TET-E-QD-S	*erm*(B)-*tet*(K)-*tet*(M)-*tet*(L)-*ant(6)-la*
13	1	*E. faecium*	TET-E-CIP-QD	*erm*(C)-*tet*(M)-*tet*(L)-*cat*(A)
14	3	*E. faecalis*	TET-E-QD-RD-S	*erm*(B)-*tet*(K)-*tet*(M)-*tet*(L)
14	4	*E. faecalis*	TET-E-CIP-C-QD	*erm*(B)-*tet*(K)-*tet*(L)-*cat*(A)-*ant*(6)-Ia
15	3	*E. faecalis*	E-QD	*erm*(C)-*tet*(K)-*tet*(L)
15	4	*E. faecalis*	QD	*erm*(C)-*tet*(K)
16	1	*E. faecium*	TET-QD-S	*erm*(C)-*tet*(M)-*tet*(L)
16	2	*E. faecium*	TET-QD-RD-S	*erm*(C)-*tet*(M)-*tet*(L)-*cat*(A)
16	3	*E. faecalis*	TET-QD-RD-S-LNZ	*tet*(M)-*tet*(L)-*cat*(A)-*ant*(6)-Ia
16	4	*E. faecalis*	TET-E-QD-RD-S	*tet*(M)-*tet*(L)-*cat*(A)-*ant*(6)-Ia
18	2	*E. faecium*	TET-E-QD-RD	*erm*(C)-*tet*(M)-*cat*(A)
21	4	*E. faecium*	TET-QD-RD-S	*tet*(M)-*tet*(L)-*cat*(A)
22	2	*E. faecalis*	TET-E-C-QD-RD-S	*erm*(B)-*tet*(K)-*tet*(M)-*tet*(L)-*cat*(A)
25	1	*E. faecium*	TET-E-S	*erm*(B)-*tet*(K)-*tet*(M)-*cat*(A)-*ant*(6)-Ia
25	2	*E. faecalis*	TET-E-QD-S	*erm*(B)-*tet*(K)-*tet*(M)-*tet*(L)-*ant*(6)-Ia
25	3	*E. faecalis*	TET-E-C-QD-S	*erm*(B)-*tet*(K)-*tet*(M)-*tet*(L)-*cat*(A)-*ant*(6)-Ia

Table S6 (Cont.). Phenotype and genotype characterization of *E. faecalis* (n=43) and *E. faecium* (n=11) isolates (continuation).

Sample	Isolated	Specie	Resistance Phenotype	Resistance Genotype
26	1	*E. faecium*	TET-E-QD-S	*erm*(B)-*erm*(C)-*tet*(K)-*tet*(M)-*tet*(L)-*cat*(A)-*ant*(6)-Ia
27	1	*E. faecium*	TET-E-QD-S	*erm*(B)-*erm*(C)-*tet*(K)-*tet*(M)-*tet*(L)-*ant*(6)-Ia
30	4	*E. faecalis*	TET-E-QD-S	*tet*(M)-*tet*(L)
31	1	*E. faecalis*	E-QD-RD-S	*tet*(L)-*ant*(6)-Ia
31	2	*E. faecalis*	TET-E-C-QD	*erm*(B)-*tet*(K)-*tet*(M)-*tet*(L)-*cat*(A)-*ant*(6)-Ia
31	3	*E. faecalis*	TET-E-C-QD	*erm*(B)-*tet*(K)-*tet*(M)-*tet*(L)
31	4	*E. faecalis*	TET-E-QD-S	*erm*(B)-*tet*(K)-*tet*(M)-*tet*(L)-*tet*(O)-*cat*(A)
32	2	*E. faecalis*	TET-E-QD-RD-S	*erm*(B)-*tet*(K)-*tet*(M)-*tet*(L)
32	3	*E. faecalis*	TET-E-QD-RD-S	*erm*(B)-*tet*(M)-*tet*(L)
32	4	*E. faecalis*	TET-E-QD-RD-S	*erm*(B)-*tet*(K)-*tet*(M)-*tet*(L)
34	1	*E. faecalis*	TET-E-QD-RD-S	*erm*(B)-*tet*(K)-*tet*(M)-*tet*(L)-*aac*(6′)-*aph*(2′′)-*cat*(A)
34	3	*E. faecalis*	TET-E-QD-RD-S	*erm*(B)-*tet*(K)-*tet*(M)-*tet*(L)-*aac(6′)-aph*(2′′)
36	1	*E. faecium*	E-QD-RD	*tet*(K)-*tet*(M)-*tet*(L)
36	2	*E. faecalis*	E-QD-RD	*tet*(L)
36	3	*E. faecalis*	VA-TET-E-C-QD-RD-S-LNZ	*erm*(B)-*tet*(K)-*tet*(M)-*tet*(L)
36	4	*E. faecalis*	TET-E-C-QD-RD-S	*erm*(B)-*tet*(K)-*tet*(M)-*tet*(L)
37	2	*E. faecalis*	TET-E-CIP-QD-F-FOS	*tet*(L)-*ant*(6)-Ia
39	2	*E. faecalis*	TET-E-QD-RD	*tet*(L)-*tet*(O)
40	4	*E. faecalis*	TET-E-C-QD-RD	*erm*(B)-*tet*(M)-*tet*(L)
